# Susceptibility to insecticides and resistance mechanisms in three populations of *Aedes aegypti* from Peru

**DOI:** 10.1186/s13071-019-3739-6

**Published:** 2019-10-22

**Authors:** Jesus Pinto, Miriam Palomino, Leonardo Mendoza-Uribe, Carmen Sinti, Kelly A. Liebman, Audrey Lenhart

**Affiliations:** 1 Instituto Nacional de Salud, Lima, Peru; 2 Instituto Nacional de Salud-Centro de Investigación en Enfermedades Tropicales “Maxime Kuczynski”-CIETROP, Iquitos, Peru; 3 American Society of Microbiology, Washington, DC USA; 4 Centers for Disease Control and Prevention, Atlanta, GA USA

**Keywords:** *Aedes aegypti*, Insecticide resistance, Peru

## Abstract

**Background:**

Epidemics of dengue, chikungunya and Zika are a growing threat to areas where *Aedes aegypti* are present. The efficacy of chemical control of *Ae. aegypti* is threatened by the increasing frequency of insecticide resistance. The objective of this study was to determine the susceptibility status as well as the biochemical and molecular mechanisms underlying insecticide resistance in three populations of *Ae. aegypti* in high risk areas of dengue, chikungunya, and Zika in Peru.

**Methods:**

Bioassays were conducted on adult *Ae. aegypti* to evaluate their susceptibility to insecticides used currently or historically for mosquito control in Peru, including six pyrethroids, three organophosphates and one organochlorine, in populations of *Ae. aegypti* from the districts of Chosica (Department of Lima), Punchana (Department of Loreto) and Piura (Department of Piura). Resistance mechanisms were determined by biochemical assays to assess activity levels of key detoxification enzyme groups (nonspecific esterases, multi-function oxidases, glutathione S-transferases and insensitive acetylcholinesterase). Real-time PCR assays were used to detect two *kdr* mutations (V1016I and F1534C) on the voltage-gated sodium channel gene.

**Results:**

Resistance to DDT was detected in all three populations, and resistance to pyrethroids was detected in all populations except the population from Chosica, which still exhibited susceptibility to deltamethrin. Resistance to organophosphates was also detected, with the exception of populations from Punchana and Piura, which still demonstrated susceptibility to malathion. In general, no increase or alteration of activity of any enzyme group was detected. Both 1016I and 1534C alleles were detected in Punchana and Piura, while only the 1534C allele was detected in Chosica.

**Conclusions:**

The results suggest that resistance to multiple classes of insecticides exist in areas important to *Ae. aegypti*-borne disease transmission in Peru. The F1534C mutation was present in all 3 populations and the V1016I mutation was present in 2 populations. To our knowledge, this is the first report of the presence of 1016I and 1534C in *Ae. aegypti* in Peru. The absence of highly elevated enzymatic activity suggests that target site resistance is a key mechanism underlying insecticide resistance in these populations, although further research is needed to fully understand the role of metabolic resistance mechanisms in these populations.

## Background

Dengue is of increasing public health concern globally. An estimated 2.5 billion people, representing 40% of the worldʼs human population, live in areas at risk of dengue transmission and there are an estimated 390 million cases of dengue per year in tropical and subtropical areas [1]. In tropical and subtropical urban areas, the mosquito *Aedes aegypti* is the main vector of dengue, chikungunya and Zika viruses. In 1947, the *Ae. aegypti* eradication programme in the Americas began, which managed to eliminate this vector in 19 countries in the region, relying heavily on the widespread application of the organochlorine insecticide DDT [2, 3]. However, the decline of this programme at the end of the 1960s led to broad *Ae. aegypti* reinfestations originating from areas that had not succeeded in fully eliminating it [4].

*Aedes aegypti* was first reported in Peru in 1852 and is believed to have entered from the north *via* Guayaquil, Ecuador, progressively establishing itself along the north and central Peruvian coast, eventually extending its distribution to Tacna in the far south [5]. *Aedes aegypti* was eliminated from Peru in 1958 but was subsequently reintroduced and detected in 1984 in Loreto and Ucayalí, and then dispersed to neighboring regions including San Martín and the central jungle (Satipo and Chanchamayo). In 1990, an explosive epidemic of dengue (caused by DENV-1) occurred in several key cities in the Peruvian Amazon, and at present, almost all areas of the country where *Ae. aegypti* are present report cases of dengue, with the circulation of all four dengue serotypes now documented in Peru [6]. *Aedes aegypti* is now widely dispersed in 20 departments nationwide and has been detected in 486 districts, resulting in over 14 million people at risk of contracting *Ae. aegypti*-borne illnesses [7].

The integrated control of *Ae. aegypti* in Peru is based principally on the use of insecticides to reduce the populations of larvae and adults. The reduction of larval populations has historically been based on the application of the organophosphate insecticide temephos to larval habitats. To reduce adult mosquito populations, the pyrethroid insecticides cyfluthrin, alpha-cypermethrin, deltamethrin, lambda-cyhalothrin and cypermethrin had been used up until 2014, when this study was conducted.

Insecticide resistance in mosquitoes can be caused by increased activity of enzymes that detoxify insecticides (‘metabolic resistance’), alterations at the target site of the insecticides in the mosquito (‘target site resistance’), thickening of the cuticle to inhibit insecticide penetration, and the development of behaviors that lead to the mosquito avoiding contact with insecticides. Metabolic resistance typically involves the increased activity of any of the three main groups of detoxification enzymes: carboxylesterases, multi-function oxidases (MFOs), and glutathione S-transferases (GSTs) [8]. Target site resistance in mosquitoes often involves alterations that render acetylcholinesterase insensitive or conformational changes on the voltage-gated sodium channel that prevent insecticide binding and thus prevents the mosquito from being knocked down after exposure (resulting in knockdown resistance, *kdr*) [9, 10]. *Kdr* is associated with resistance to both the organochlorine insecticide DDT and pyrethroid insecticides, and results from non-synonymous point mutations on the gene that codes for the voltage-gated sodium channel [10]. Pyrethroid insecticides act at the level of the voltage-gated sodium channel (VGSC), which is a transmembrane protein that is made up of four homologous domains (I-IV), with each domain consisting of six hydrophobic segments (S1-S6) [11]. Several mutations have been identified at different positions on the VGSC in *Ae. aegypti* (V410L, S989P, I1011M/V, V1016G/I, F1269C, F1534C) [12–15], with V1016I/G and F1534C occurring on IIS6 and IIIS6, respectively, clearly associated with pyrethroid resistance [13, 16].

Widespread vector control for malaria in Peru began in the 1960s with the use of DDT. Organophosphates (fenitrothion and chlorpyrifos) were also used in the 1980s, and in the 1990s pyrethroid use became common (cyfluthrin, alpha-cypermethrin, cypermethrin and lambda-cyhalothrin) [17]; during this same period, dengue vector control activities were first started. Deltamethrin was first used for *Ae. aegypti* control in 2000. National surveillance data from Peru’s National Institute of Health shows that insecticide resistance in *Ae. aegypti* was first detected in 2005 in the departments of Tumbes (to permethrin, propoxur and carbaryl) and La Libertad (to propoxur and bendiocarb). In 2011, resistance to alpha-cypermethrin was reported in the department of Madre de Dios and deltamethrin resistance was detected in the department of Piura. Although routine resistance monitoring is carried out by Peru’s National Institute of Health, very little is known about the underlying mechanisms driving the resistance. The objective of the present study was to link susceptibility status with biochemical and molecular mechanisms involved in resistance in key populations of *Ae. aegypti* from three departments in Peru.

## Methods

### Study areas

**Fig. 1:**
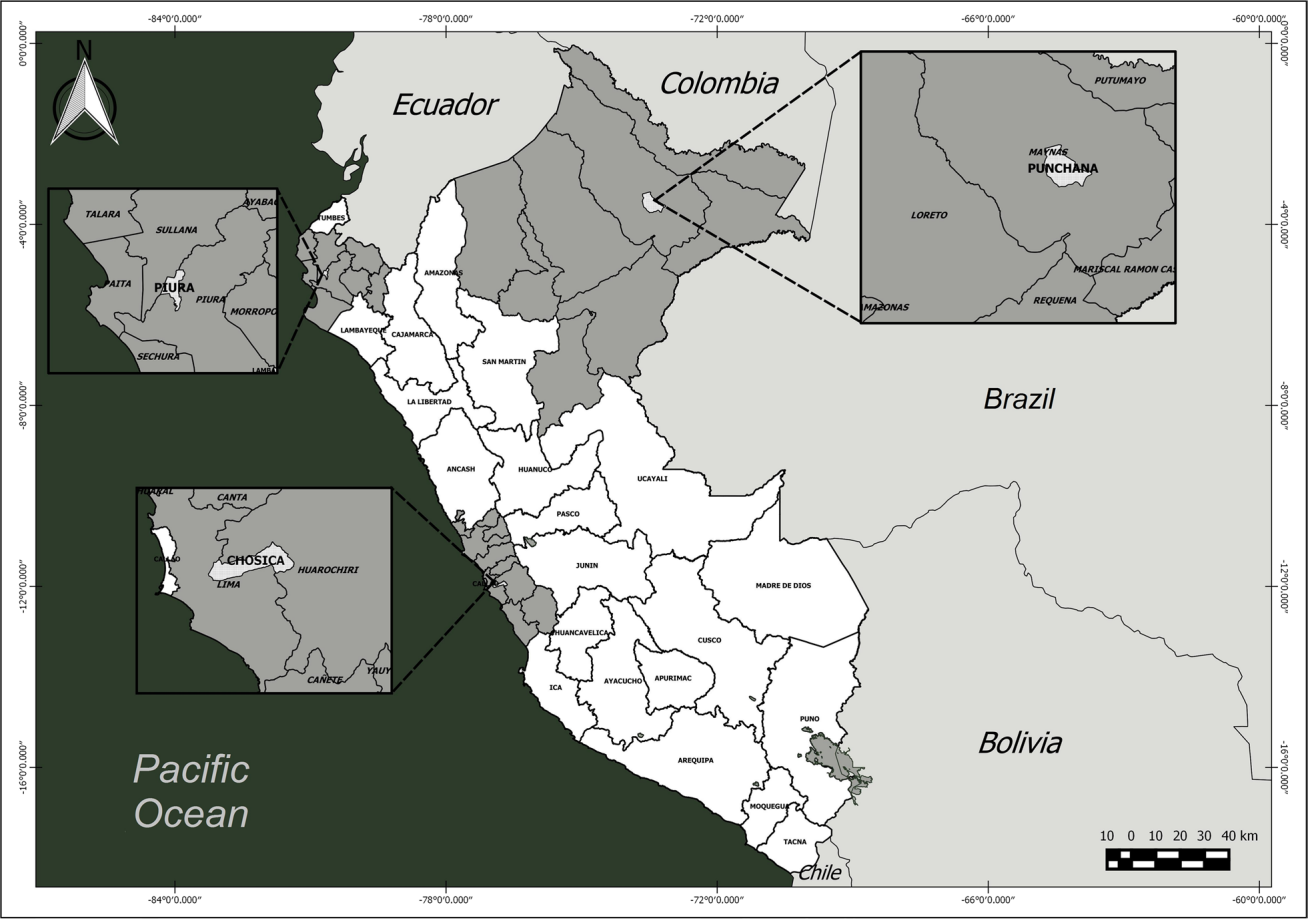
Map of Peru, showing the sites where *Aedes aegypti* eggs were collected

The study was conducted in three departments in Peru (Lima, Loreto and Piura) that represent two ecological regions of the country: the coast and the Amazon. The Peruvian coastline is formed by a desert strip that extends from north to south and borders the Pacific Ocean to the west and is divided into three zones: the north coast that borders Ecuador (where the department of Piura is located); the central coast where the department of Lima is located; and the south coast that runs to the border with Chile. The department of Loreto is located in the Amazon region of central Peru (Fig. 1).

In Lima department, the city of Chosica (11°56′41″S, 76°41′44.7″W) was selected, with a population of 218,976 inhabitants [18] and an area of 236.5 km^2^. The city of Lima (capital of Lima department and capital of Peru) experienced an outbreak of dengue in 2005, with 440 cases [6]. This outbreak was of particular interest because until that time, *Ae. aegypti* had not been commonly reported in Lima. In Piura, the districts of Piura and Castilla (5°14′56.2″S, 80°37′32.9″W) were selected; together these districts have 459,176 inhabitants [18] and an area of 992.5 km^2^. Since the 1990s, Piura has experienced endemic dengue transmission, with a large outbreak occurring in 2001 with 11,713 cases. In Loreto, the Punchana district of the city of Iquitos was selected (3°42′13.1″S, 73°14′57″W) which has 69,068 inhabitants [18] and an area of 1,573.4 km^2^. The city of Iquitos has also experienced endemic dengue transmission for more than two decades, and in 2011, experienced a large and severe outbreak with 21,245 cases and 19 deaths [19].

### Entomological collections

Between February and August of 2014, a cross-sectional entomological survey was conducted using oviposition traps (‘ovitraps’) that consisted of 500 ml water-holding cups lined with a strip of filter paper as an oviposition substrate. The ovitraps were distributed every 5 blocks in both residential and non-residential sites throughout the sampling area. Data on temperature, humidity and the coordinates of each ovitrap were recorded. The ovitraps were left *in situ* for up to three weeks and then collected. The ovitrap papers were transferred to the National Entomology Reference Laboratory at Peru’s National Institute of Health (INS), Lima, for egg detection and positive papers were placed in a humid chamber for 7 days, to favor their maturation. The papers were stored at 25 °C and 70% humidity for no more than three months before hatching. The paper strips containing the eggs were immersed in water to allow the larvae to hatch (generation F_0_). The larvae and resulting adults were reared and bred under controlled conditions of humidity (80 ± 10% RH) and temperature (27 ± 1 °C) in the insectary of the INS National Entomology Reference Laboratory, Lima, Peru. All bioassays, biochemical and molecular tests were conducted using F_1_ progeny of the generation that emerged from the original field-collected eggs.

### Insecticide bioassays

For adult mosquito bioassays, insecticides chosen were those used over the past 20 years in Peru for vector control, including DDT, organophosphates, carbamates and pyrethroids. Bioassays were performed following the standard protocol developed by the WHO [20], in which mosquitoes were exposed to insecticide-impregnated papers (University of Malaysia Vector Control Research Unit, Universiti Sains Malaysia). An average of 300 unfed female mosquitoes aged 3 to 5 days-old from each population were evaluated for susceptibility to each insecticide. In addition, an average of 100 mosquitoes from the Rockefeller susceptible strain were evaluated simultaneously with each of the populations and for each insecticide, to verify the insecticidal effects of the impregnated papers. Mosquitoes were exposed for 1 h to papers treated with alpha-cypermethrin (0.05%), cypermethrin (0.05%), deltamethrin (0.05%), etofenprox (0.5%), lambda-cyhalothrin (0.05%), permethrin (0.75%), DDT (4%), malathion (5%) and pirimiphos-methyl (0.25%), and exposed for 2 h to papers treated with fenitrothion (1%). Negative controls were also included in which mosquitoes were exposed to papers impregnated with solvents only. Following exposure, mosquitoes were transferred to holding tubes and provided with 10% sugar solution. Mortality was recorded at 24 h post-exposure. All bioassays were performed in the INS National Entomology Reference Laboratory.

### Biochemical tests

All biochemical analyses were performed based on Centers for Disease Control and Prevention (CDC) protocols [21]. An average of 120 adult females per field population and 120 adult females from the Rockefeller susceptible strain aged 3 to 5 days were killed by freezing and stored at − 20 °C for a maximum of two months prior to processing. The head and thorax of each mosquito were separated from the abdomen and homogenized in 100 μl of potassium phosphate (KPO_4_) buffer (6.6 g of dibasic potassium phosphate/1.7 g of monobasic phosphate/100 ml of distilled water, pH 7.2). The homogenate was diluted to 1 ml using the same buffer.

A volume of 100 μl of homogenate was evaluated in triplicate for each mosquito. Homogenates were transferred to a 96-well flat bottom microplate, with 30 mosquitoes analyzed in triplicate per plate. The Rockefeller strain was used as the susceptible reference population in all biochemical analyses. The enzyme groups evaluated were: beta-type non-specific esterases (NSEs), MFOs, GSTs, and insensitive or modified acetylcholinesterase (MACE) [22–24]. The substrates used in each assay included: β-naphthyl acetate for NSE, reduced glutathione and CDNB (1-chloro-2,4′-dinitrobenzene) for GSTs, and TMBZ (3,3′, 5,5′-tetramethylbenzidine dihydrochloride) for MFO. Acetylthiocholine iodide as well as the carbamate insecticide propoxur were used to measure acetylcholinesterase insensitivity. The positive controls consisted of cytochrome C for MFO and β-naphthol for NSE and were run in triplicate on the corresponding plates, and the three negative controls per plate consisted of the potassium phosphate buffer. The total protein concentration for each mosquito was determined using the methodology described by Brogdon [25, 26]. This allowed for the detection of differences in the body masses of the mosquitoes, which was used as a correction factor for the enzymatic analyses. Absorbances were measured by spectrophotometry using a microplate reader (Multiskan GO, Thermo Fisher Scientific, Hampton, NH, USA), at the wavelengths indicated for each enzyme family (540 nm for NSE, 620 nm for MFO and protein, 340 nm for GST, and 414 nm for MACE).

### Molecular analyses

Molecular analyses were performed on mosquitoes surviving the bioassays with pyrethroids and DDT, to screen for the presence of the *kdr* mutations V1016I and F1534C. DNA was extracted from individual mosquitoes by placing each adult mosquito in a PCR tube containing 50 μl of a 1:10 dilution of 10× buffer (Promega, Madison, WI, USA) containing 15 mM MgCl_2_, 50 mM KCl, and 10 mM Tris-HCl (pH 9.0 at 25 °C). The tubes were incubated at 95 °C for 10 min. The resulting DNA was stored at − 20 °C until PCR analysis.

DNA was analyzed by real-time PCR followed by a melting curve analysis based on the methodologies described by Saavedra-Rodriguez et al. [13] and Yanola et al. [27]; primer sequences are presented in Table 1. The PCR for the V1016I mutation was carried out in a total reaction volume of 20 μl using 2 μl of DNA template, 8 μl of SuperMix Green SYBR® Perfecta 2× (which contains a mixture of dNTPs, MgCl_2_, AccuStart Taq DNA polymerase, SybrGreen), 0.2 μM of the common primer Ile1016r and 0.2 μM and 0.17 μM of the specific primers Ile1016f and Val1016f, respectively. The cycling conditions were: 95 °C for 3 min followed by 35 cycles of 95 °C for 10 s, 60 °C for 10 s and 72 °C for 30 s, and a final extension step of 95 °C for 10 s. The melting curve was determined using a gradient from 65 °C to 95 °C with an increase of 0.2 °C every 10 s, where a single peak at 76 °C corresponded to a mutant homozygote (I1016/I1016), peaks at both 76 °C and 83 °C corresponded to a heterozygote (V1016/I1016) and a single peak at 83 °C corresponded to a wild type homozygote (V1016/V1016). The PCR for the F1534C mutation was carried out in a total reaction volume of 20 μl using 2 μl of DNA template, 9 μl of PerfeCTa SYBR Green SuperMix (Quantabio, Beverly, MA, USA), 0.3 μM of the common primer Phe1534+r and 0.3 μM and 0.325 μM of the specific primers Phe1534+f and Cys1534+f, respectively. The cycling conditions were: 95 °C for 3 min followed by 37 cycles of 95 °C for 10 s, 57 °C for 30 s and 72 °C for 30 s, and a final extension at 95 °C for 10 s. The melting curve was determined using a gradient from 65 °C to 95 °C with an increase of 0.5 °C every 5 s, where a single peak at 82 °C corresponded to a mutant homozygote (C1534/C1534), peaks at both 78 °C and 82 °C corresponded to a heterozygote (F1534/C1534) and a single peak at 78 °C corresponded to a wild type homozygote (F1534/F1534). The amplification and melting curve analysis were performed using a Bio-Rad CFX96 real time PCR system (Bio-Rad, Hercules, CA, USA). DNA from of *Ae. aegypti* individuals from the Rockefeller strain served as a wild-type control, while DNA from previously genotyped individuals was used for positive controls for the *kdr* mutations.

**Table 1:** Primer sequences for real-time PCR detection of *kdr* mutations V1016I and F1534C

Primer	Primer sequence (5’-3’)
Val1016f	GCGGGCGGCGGGGGCGGGGCCACAAATTGTTTCCCACCCGCACCGG
Ile1016f	GCGGGCACAAATTGTTTCCCACCCGCACTGA
Ile1016r	TGATGAACCSGAATTGGACAAAAGC
Cys1534+f	GCGGGCAGGGCGGCGGGGGCGGGGCCTCTACTTTGTGTTCTTCATCATGTG
Phe1534+f	GCGGGCTCTACTTTGTGTTCTTCATCATATT
Phe1534+r	TCTGCTCGTTGAAGTTGTCGAT

### Data analysis

The results of the bioassays were interpreted based on the percent mortality at 24 h post-exposure to each insecticide, using the criteria recommended by WHO: 98–100% mortality indicates susceptibility; 90–97% mortality suggests that resistance may be developing; and < 90% mortality indicates the presence of resistance [20].

For the biochemical analyses, mean absorbance, standard deviations and coefficients of variation were calculated based on the data from the three replicates of each mosquito. Samples with a coefficient of variation higher than 0.10 were not included in the analyses. The mean absorbance of the negative control was subtracted from the value of each sample for each enzyme to eliminate background noise. Corrections were performed using the total protein values because there were significant differences in body size among the individuals. Cut-off values for each enzyme group were determined using the values obtained from the Rockefeller susceptible reference strain of *Ae. aegypti* by adding the average of the optical density results to twice the standard deviation of the replicates. Averages of the optical densities of the field populations greater than this cut-off value were considered elevated for the enzyme group in question. In addition, the average absorbances of each population for each of the enzyme groups were analyzed to determine whether they reflected a normal distribution using the Shapiro-Wilk test. To determine the differences in enzymatic activity in each population, the enzymatic activity profiles of each population were compared with the Rockefeller strain using the Kruskall-Wallis non-parametric test.

The allelic frequencies for 1016I and 1534C were calculated using the following equation:

$$ \frac{{{\text{No}}.{\text{ of heterozygotes }} + { 2 }\left( {{\text{No}}.{\text{ of homozygotes}}} \right)}}{{ 2 { }\left( {{\text{Total no}}.{\text{ of mosquitoes analyzed}}} \right)}} $$

## Results

### Insecticide bioassays

**Fig. 2:**
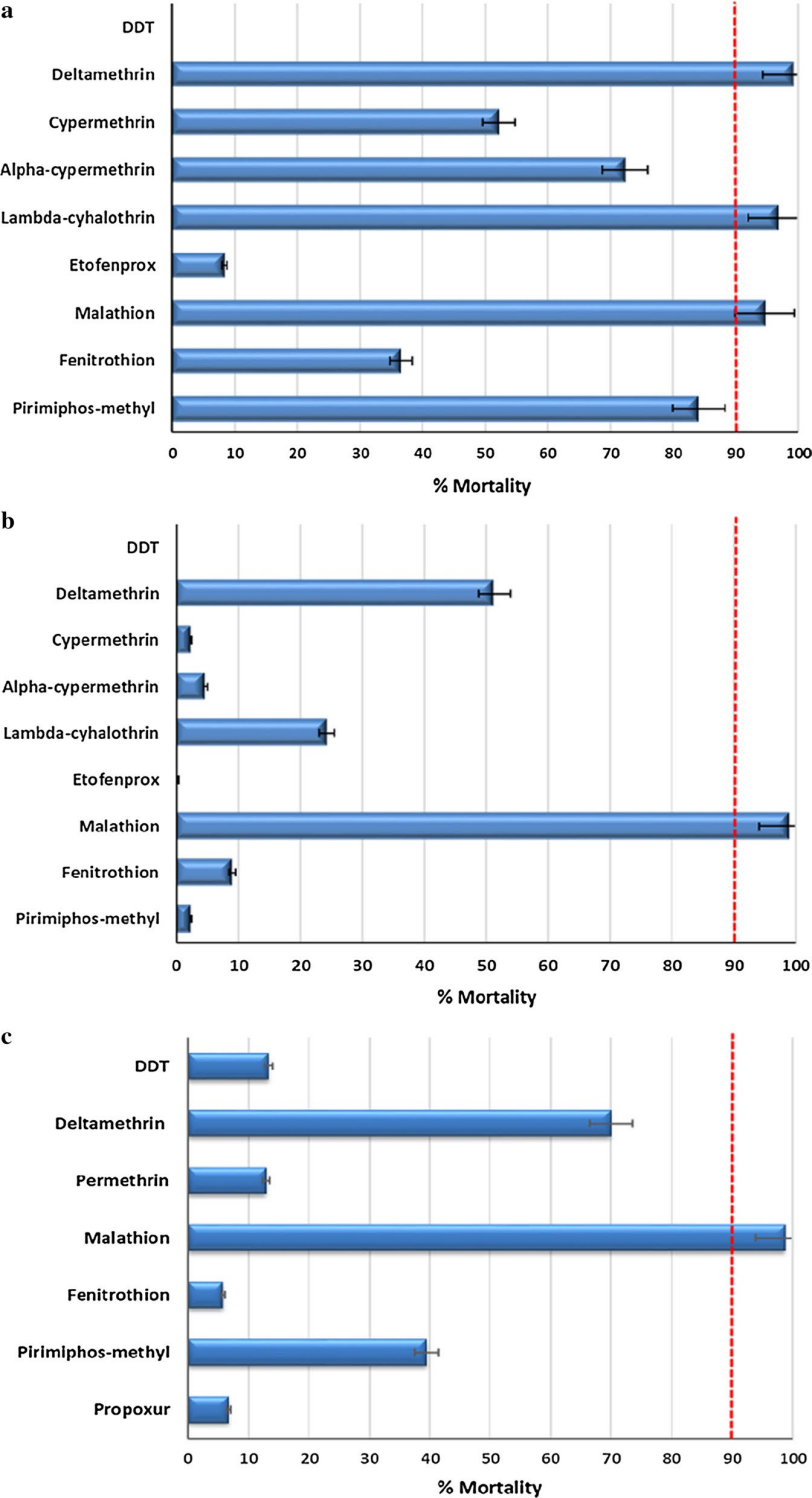
Bioassay results from Peruvian *Ae. aegypti* from Chosica (**a**), Punchana (**b**), and Piura (**c**). Red dashed line shows the threshold value of 90%, below which a population is considered insecticide-resistant

A total of 1724 eggs were collected from the field in Chosica, 2122 eggs in Punchana and 899 eggs in Piura. Bioassays were conducted using *Ae. aegypti* F_1_ progeny from Chosica (*n* = 2693), Punchana (*n* = 2702) and Piura (*n* = 2396). Table 2 and Fig. 2 show the average percentages of mortality for the insecticides tested for all three populations. Mortality results from the Chosica population revealed: (i) resistance to alpha-cypermethrin, cypermethrin, etofenprox, DDT, fenitrothion and pirimiphos-methyl; (ii) developing resistance to lambda-cyhalothrin and malathion, and (iii) susceptibility to deltamethrin. The Punchana and Piura populations showed resistance to all evaluated insecticides with the exception of malathion. Mortality in the controls was always less than 5%, so Abbott’s formula for corrected mortality was not applied.

**Table 2:** Results of bioassays conducted on Peruvian *Aedes aegypti*

Insecticide	Population	No. tested	% Mortality^a^	95% CI	Resistance status
DDT	Chosica	299	0	–	R
	Punchana	300	0	–	R
	Piura	301	13.3 ± 9.2	7.4–19.1	R
Deltamethrin	Chosica	294	99.3 ± 1.6	98.3–100.3	S
	Punchana	298	51.3 ± 2.9	43.2–59.6	R
	Piura	604	74.7 ± 0.4	70.2–79	R
Cypermethrin	Chosica	301	52.2 ± 0.1	39.8–65.3	R
	Punchana	302	2.3 ± 2.7	0.6–4.0	R
	Piura	nt	nt	–	nt
Alpha-cypermethrin	Chosica	308	72.4 ± 3.4	63.6–80.6	R
	Punchana	295	4.7 ± 5.0	1.6–8.0	R
	Piura	nt	nt	–	nt
Lambda-cyhalothrin	Chosica	290	96.9 ± 5.2	93.6–100.2	RV
	Punchana	301	24.3 ± 5.5	20.8–27.8	R
	Piura	nt	nt	–	nt
Etofenprox	Chosica	302	8.3 ± 10.3	1.7–14.8	R
	Punchana	300	0.3 ± 1.2	-0.4–1.1	R
	Piura	nt	nt	–	nt
Permethrin	Chosica	nt	nt	–	nt
	Punchana	nt	nt	–	nt
	Piura	302	12.9 ± 6.3	8.9–16.9	R
Malathion	Chosica	301	94.7 ± 4.3	92–97.4	RV
	Punchana	304	99.0 ± 1.8	97.9–100.1	S
	Piura	586	98.8 ± 2.2	97.8–99.7	S
Fenitrothion	Chosica	303	36.6 ± 7.9	31.7–41.7	R
	Punchana	301	9.0 ± 6.9	4.7–13.5	R
	Piura	309	5.8 ± 5.0	2.8–9.1	R
Pirimiphos-methyl	Chosica	295	84.1 ± 7.1	79.57–8.54	R
	Punchana	301	2.3 ± 2.7	0.6–4.0	R
	Piura	294	39.5 ± 9.2	33.7–45.4	R

^a^Values are the mean ± SD. A minimum of three assays were performed per insecticide at each site

*Abbreviations*: CI, confidence interval; nt, not tested; R, resistant; S, susceptible; RV, resistance to be verified

### Biochemical tests

**Fig. 3:**
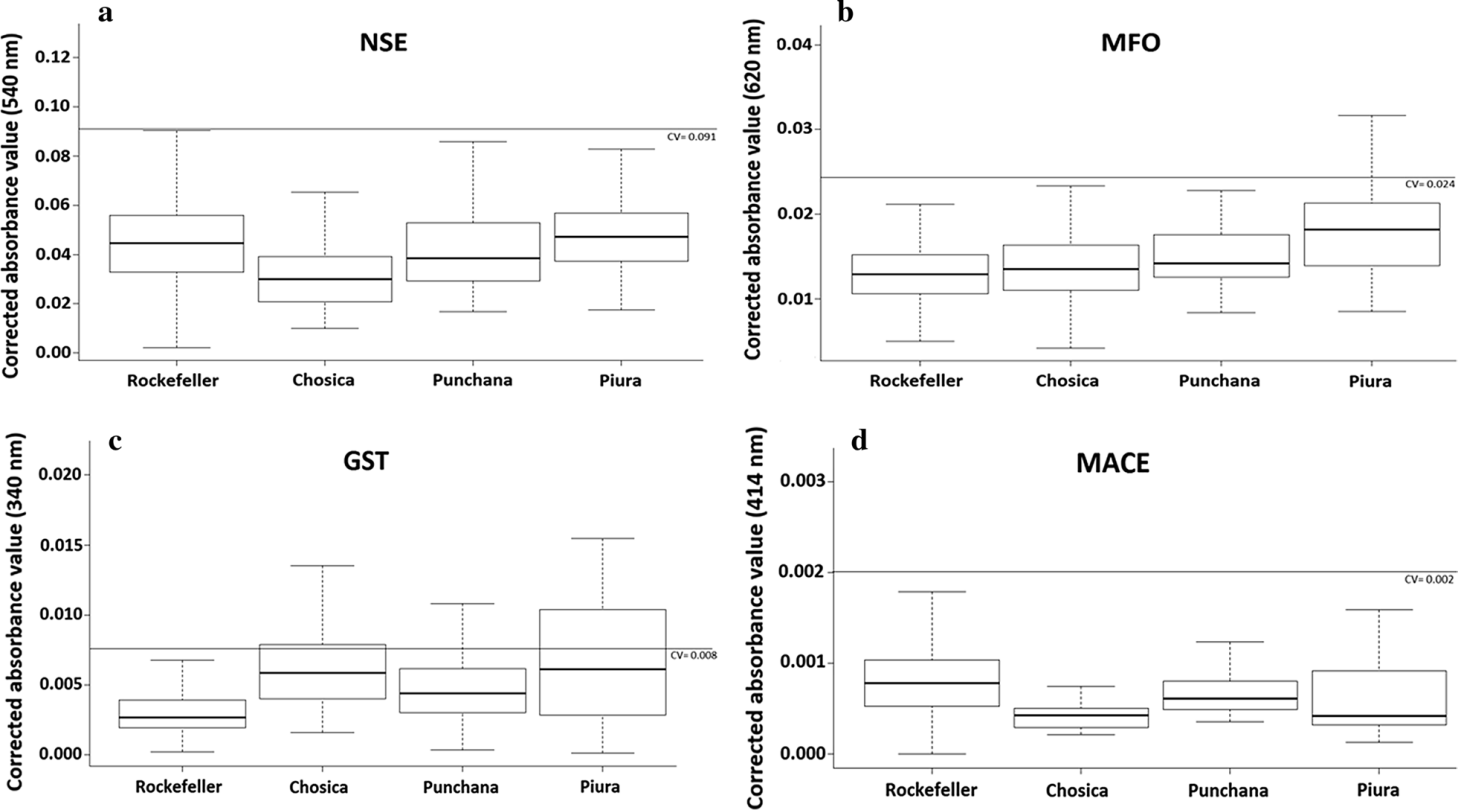
Box plots of the corrected mean absorbances for the biochemical tests for non-specific esterases (NSE) (**a**), mixed function oxidases (MFO) (**b**), glutathione s-transferases (GST) (**c**), and modified acetylcholinesterase (MACE) (**d**). The horizontal gray line represents the cut-off threshold, above which a population would be considered to have elevated activity of that enzyme group. Susceptible laboratory colony (Rockefeller) shown for comparison

The distribution of absorbances reflecting the levels of enzymatic activity detected in the three populations of *Ae. aegypti*, compared to the Rockefeller reference strain is shown in Fig. 3. In general, no increased or altered of activity of any enzyme group was detected since the absorbance averages were below their cut-off values. However, it should be noted that while the population from Piura presented an overall average absorbance below the cut-off value for MFO (average absorbance = 0.0202; cut-off = 0.0243), a notable proportion (18%) of individuals showed increased levels of activity of these enzymes. Similarly, the populations from Chosica and Piura showed overall average absorbances for GSTs that were below the cut-off value (average absorbances = 0.0062 and 0.0077, respectively; cut-off = 0.0085), yet showed elevated GST activity in a sizable proportion of individuals (18% and 38%, respectively).

### Molecular analyses

In the survivors of the DDT and pyrethroid bioassays, the *kdr* alleles 1016I and 1534C were present with overall frequencies of 0.42 and 0.84, respectively. The V1016I mutation was only detected in the populations from Punchana and Piura, with allele frequencies ranging from 0.42 to 0.72 (Table 3). The F1534C mutation was detected in all three populations with allele frequencies ranging from 0.47 to 1.0, with the homozygous mutant genotype predominating in the Punchana and Piura populations (Table 4). When comparing across the survivors of exposure to different insecticides, no differences in allele frequencies were detected based on insecticide type.

**Table 3:** V1016I allele frequencies and genotypes by population and survivors of insecticide exposure: VV, Val1016/V1016 wild type; VI, V1016/I1016 heterozygote; and II, I1016/I1016 homozygote mutant

Population	Insecticide	Total	VV	VI	II	Allele frequency		Genotype frequency		
						V1016	I1016	V1016/V1016	V1016/I1016	I1016/I1016
Chosica	Alpha-cypermethrin	34	34	0	0	1.00	0.00	1.00	0.00	0.00
	Cypermethrin	32	32	0	0	1.00	0.00	1.00	0.00	0.00
	Deltamethrin	5	5	0	0	1.00	0.00	1.00	0.00	0.00
	Etofenprox	35	35	0	0	1.00	0.00	1.00	0.00	0.00
	DDT	31	31	0	0	1.00	0.00	1.00	0.00	0.00
Punchana	Alpha-cypermethrin	33	7	16	10	0.45	0.55	0.21	0.48	0.30
	Cypermethrin	33	5	13	15	0.35	0.65	0.15	0.39	0.45
	Deltamethrin	37	1	19	17	0.28	0.72	0.03	0.51	0.46
	Etofenprox	32	9	13	10	0.48	0.52	0.28	0.41	0.31
	Lambda-cyhalothrin	33	5	18	10	0.42	0.58	0.15	0.55	0.30
	Permethrin	33	4	16	13	0.36	0.64	0.12	0.48	0.39
	DDT	31	5	15	11	0.40	0.60	0.16	0.48	0.35
Piura	Alpha-cypermethrin	28	3	17	8	0.41	0.59	0.11	0.61	0.29
	Cypermethrin	29	4	19	6	0.47	0.53	0.14	0.66	0.21
	Deltamethrin	29	7	16	6	0.52	0.48	0.24	0.55	0.21
	Etofenprox	30	7	19	4	0.55	0.45	0.23	0.63	0.13
	Lambda-cyhalothrin	30	7	14	9	0.47	0.53	0.23	0.47	0.30
	Permethrin	32	7	23	2	0.58	0.42	0.22	0.72	0.06
	DDT	30	8	19	3	0.58	0.42	0.27	0.63	0.10

**Table 4:** F1534C allele frequencies and genotypes by population and survivors of insecticide exposure: FF = F1534/F1534 wild type, FC = F1534/C1534 heterozygote, CC = C1534/C1534 homozygote mutant

Population	Insecticide	Total	FF	FC	CC	Allele frequency		Genotype frequency		
						F1534	C1534	F1534/F1534	F1534/C1534	C1534/C1534
Chosica	Alpha-cypermethrin	35	6	22	7	0.49	0.51	0.17	0.63	0.20
	Cypermethrin	32	7	18	7	0.50	0.50	0.22	0.56	0.22
	Deltamethrin	5	1	0	4	0.20	0.80	0.20	0.00	0.80
	Etofenprox	35	7	23	5	0.53	0.47	0.20	0.66	0.14
	DDT	31	5	18	8	0.45	0.55	0.16	0.58	0.26
Punchana	Alpha-cypermethrin	33	0	0	33	0.00	1.00	0.00	0.00	1.00
	Cypermethrin	33	0	0	33	0.00	1.00	0.00	0.00	1.00
	Deltamethrin	37	0	0	37	0.00	1.00	0.00	0.00	1.00
	Etofenprox	32	0	0	32	0.00	1.00	0.00	0.00	1.00
	Lambda-cyhalothrin	33	0	0	33	0.00	1.00	0.00	0.00	1.00
	Permethrin	33	0	0	33	0.00	1.00	0.00	0.00	1.00
	DDT	31	0	0	31	0.00	1.00	0.00	0.00	1.00
Piura	Alpha-cypermethrin	28	0	0	28	0.00	1.00	0.00	0.00	1.00
	Cypermethrin	31	0	0	31	0.00	1.00	0.00	0.00	1.00
	Deltamethrin	29	0	0	29	0.00	1.00	0.00	0.00	1.00
	Etofenprox	30	0	0	30	0.00	1.00	0.00	0.00	1.00
	Lambda-cyhalothrin	29	0	0	29	0.00	1.00	0.00	0.00	1.00
	Permethrin	32	0	0	32	0.00	1.00	0.00	0.00	1.00
	DDT	31	1	0	30	0.03	0.97	0.03	0.00	0.97

## Discussion

Since 1995, the INS National Entomology Reference Laboratory and its network of regional laboratories have been monitoring insecticide resistance in various species of *Anopheles* and *Ae. aegypti*. In *Anopheles albimanus*, evidence of widespread resistance to insecticides has been reported since 1998 [17], and over the past 10 years, *Ae. aegypti* has demonstrated a loss of susceptibility, mainly to pyrethroids. Results from the present study of *Ae. aegypti* from Chosica, Punchana and Piura showed high levels of resistance to DDT, multiple pyrethroids (with the exception of deltamethrin in Chosica), and organophosphates (with the exception of malathion in Punchana and Piura). The resistance to multiple pyrethroids as well as DDT could be at least partially conferred by the *kdr* mutations V1016I and F1534C, which were detected in individuals from these populations that survived bioassays, with the notable absence of 1016I in Chosica. According to Du et al. [28], several mutations on the voltage gated sodium channel gene have been associated with pyrethroid resistance in *Ae. aegypti*, with V1016I and F1534C having been repeatedly detected in resistant populations in the Americas. However, given that no clear patterns emerged regarding allele frequencies and survivorship to different insecticides, we cannot draw any conclusions regarding any differential contributions that the *kdr* alleles may have in conferring resistance to the different pyrethroids tested.

Interestingly, no significant associations were found between population-level insecticide resistance phenotypes and altered activity of detoxifying enzymes as analyzed in the biochemical tests. In this regard, Muthusamy and colleagues in 2015 [29] pointed out that although biochemical tests can provide an estimate of total enzymatic activity for large enzyme groups such as oxidases, it is possible that specific P450s may be involved as resistance mechanisms and may not always be detected by biochemical tests. For example, Djouaka et al. [30] conducted a microarray analysis that suggested multiple P450 genes were involved as pyrethroid resistance mechanisms in *An. gambiae* despite previously collected biochemical data that had not shown elevated levels of oxidase activity. Our bioassay and *kdr* results are comparable to those reported by Goidin et al. [31] from six sites in the Saint Martin and Guadaloupe Islands, where they also detected overexpression of several specific genes that suggested an important role in metabolic resistance, including GSTe2, CCEae3a, CYP6BB2, CYP6M11 and CYP9J23. Although on a population scale we did not detect altered enzyme activities, notable proportions of individuals from Piura (for both MFOs and GSTs) and Chosica (for GSTs) did show elevated activity, which could be an indication of resistance mechanisms stemming from a few specific genes within each of those larger enzyme families. However, further molecular analyses would be required to determine if this is the case.

High frequency of resistance to DDT was found in all three of the evaluated populations, with bioassay mortalities ranging from 0–13.3%. Since DDT has not been used in Peru for vector control in over 20 years, the ongoing resistance could be the result of cross-resistance with pyrethroids due to shared modes of action. With respect to this finding, Bisset et al. [32], previously reported resistance to both DDT and lambda-cyhalothrin in *Ae. aegypti* in Trujillo (department of La Libertad, Peru) and resistance to DDT, beta-cypermethrin, deltamethrin and lambda-cyhalothrin in a population from the city of Tumbes (Tumbes department, Peru), hypothesizing that resistance to pyrethroids could be the result of cross-resistance to DDT through the *kdr* mechanism. However, the high frequency of DDT resistance observed in our study is likely not only due to *kdr*, particularly given that in Chosica mortality to DDT was 0% yet there was a complete absence of the 1016I *kdr* allele. Similar levels of resistance to DDT have been reported in other countries in the Americas including Colombia, Trinidad, Puerto Rico, Jamaica, Haiti, Dominican Republic, Venezuela and Suriname [33–35]. In Colombia, Fonseca et al. [33] evaluated the susceptibility status of *Ae. aegypti* in twelve mosquito populations and found that all populations were resistant to DDT and had elevated levels of GST activity and showed overexpression of the *GSTe2* gene. Ocampo et al. [34] reported that resistance to DDT, found in populations of *Ae. aegypti* from 10 localities of Colombia, was genetically fixed since reinfestation occurred with populations that were already resistant to DDT. Further analyses can help to elucidate the roles that metabolic enzymes might be playing in maintaining resistance to DDT, particularly given that signs of elevated GST activity were detected in Chosica and Piura.

Although the populations of *Ae. aegypti* from Punchana and Piura were susceptible to malathion, the *Ae. aegypti* from Chosica presented incipient resistance (94.7% mortality). This is worrisome, given that in 2014 malathion was introduced for the widespread control of *Ae. aegypti* in Peru after extensive resistance to cypermethrin was detected in more than 13 regions of Peru (routine surveillance data, INS). Similarly, Yadav et al. [36] studied *Stegomyia albopicta* and *St. aegypti* in India and found general susceptibility to malathion despite its use to control mosquito-borne disease outbreaks; however, delayed knock-down values suggested that resistance to malathion was developing in the study area. In contrast, Arslan et al. [37] detected resistance to DDT, bendiocarb, permethrin and malathion in a study of *Ae. aegypti* and *Ae. albopictus* in Pakistan, despite a lack of use of organophosphates and carbamates for vector control. The authors suggested that the resistance could be due to the routine use of pesticides from these classes in agriculture in and around the study areas. This highlights the importance of monitoring vector populations for susceptibility to a broad spectrum of insecticides, as selective pressures for insecticide resistance may not always originate from public health interventions.

## Conclusions

The populations of *Ae. aegypti* from Chosica, Piura and Punchana showed resistance to multiple classes of insecticides, with high levels of resistance to DDT, pyrethroids and organophosphates with the exception of malathion, which is one of the few remaining alternatives for the control of *Ae. aegypti* in Peru. The mechanisms associated with this resistance included the *kdr* mutations F1534C and V1016I, but biochemical tests did not conclusively detect elevated activity in any large detoxification enzyme families. Resistance to insecticides can threaten the efficacy of vector-borne disease control, and the results reported here highlight the importance of routine monitoring of insecticide resistance because they suggest that resistance to malathion could be developing in Peru. In this context, it is necessary to understand which alternative insecticides would be most effective for the control of *Ae. aegypti* and how the resistance that has already been detected can be effectively managed, especially given the ever-increasing public health burden due to *Ae. aegypti*-borne arboviruses in Peru.

## Data Availability

All data generated or analyzed during this study are included in this published article.

## Abbreviations

MFOs: multi-function oxidases
GSTs: glutathione S-transferases
MACE: modified acetylcholinesterase
